# Supplementary material for: Mutations in PCYT2 disrupt etherlipid biosynthesis and cause a complex hereditary spastic paraplegia
Source: Brain. 2019 Oct 22;142(11):3382–97. doi: 10.1093/brain/awz291 (PMC6821184; doi:10.1093/brain/awz291)
Supplement: awz291_Supplementary_Data [file awz291_supplementary_data.zip › awz291-Suppl_data/Supplementary_Data4.zip!/Supplementary information 4 - plasma lipidomics.zip!/Plasma lipidomics/Control vs Patient/Statistical plots and data/TG species/Random forest.pdf]

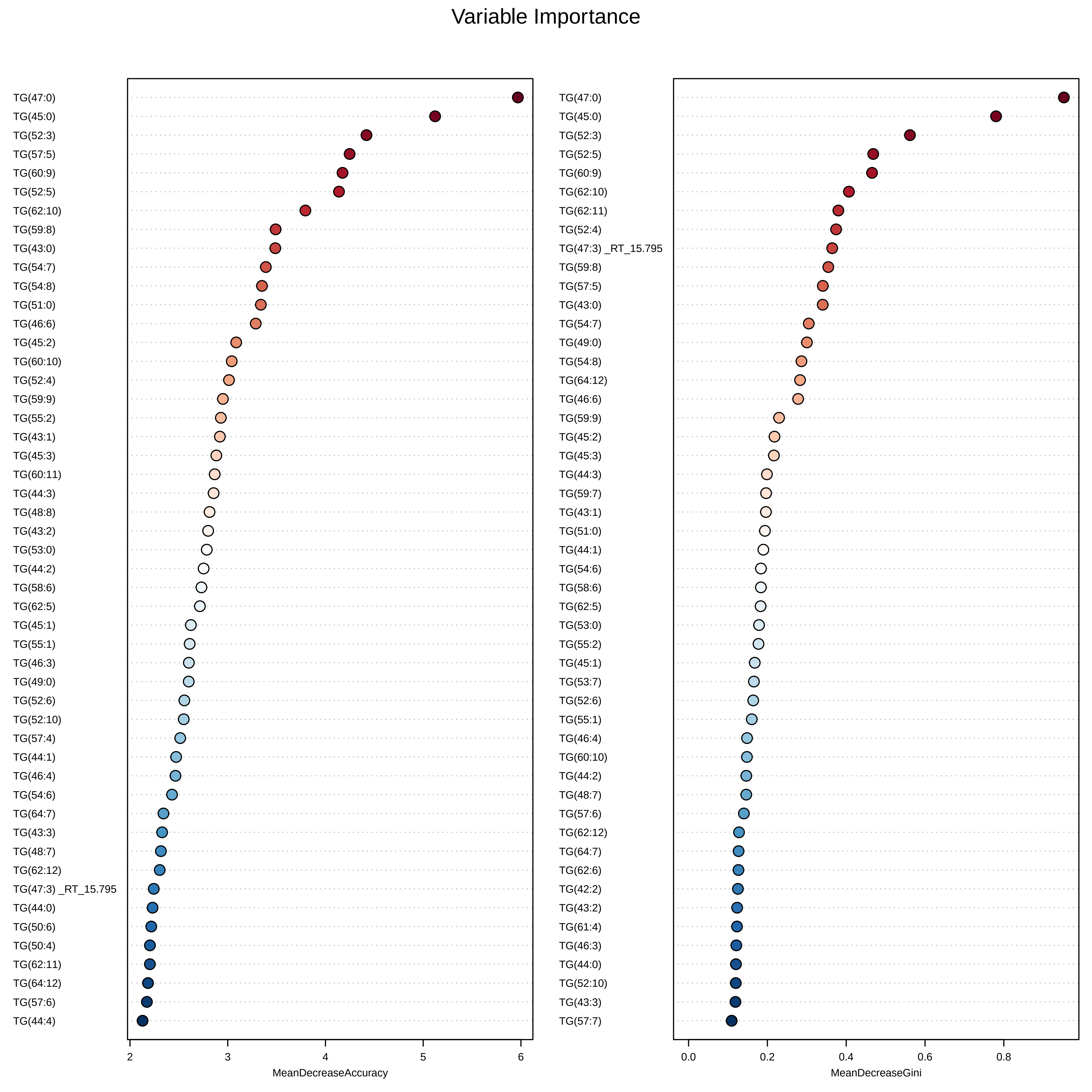
Variable Importance
TG(47:0)
TG(45:0)
TG(52:3)
TG(57:5)
TG(60:9)
TG(52:5)
TG(62:10)
TG(59:8)
TG(43:0)
TG(54:7)
TG(54:8)
TG(51:0)
TG(46:6)
TG(45:2)
TG(60:10)
TG(52:4)
TG(59:9)
TG(55:2)
TG(43:1)
TG(45:3)
TG(60:11)
TG(44:3)
TG(48:8)
TG(43:2)
TG(53:0)
TG(44:2)
TG(58:6)
TG(62:5)
TG(45:1)
TG(55:1)
TG(46:3)
TG(49:0)
TG(52:6)
TG(52:10)
TG(57:4)
TG(44:1)
TG(46:4)
TG(54:6)
TG(64:7)
TG(43:3)
TG(48:7)
TG(62:12)
TG(47:3) _RT_15.795
TG(44:0)
TG(50:6)
TG(50:4)
TG(62:11)
TG(64:12)
TG(57:6)
TG(44:4)
2
3
4
5
6
MeanDecreaseAccuracy
TG(47:0)
TG(45:0)
TG(52:3)
TG(52:5)
TG(60:9)
TG(62:10)
TG(62:11)
TG(52:4)
TG(47:3) _RT_15.795
TG(59:8)
TG(57:5)
TG(43:0)
TG(54:7)
TG(49:0)
TG(54:8)
TG(64:12)
TG(46:6)
TG(59:9)
TG(45:2)
TG(45:3)
TG(44:3)
TG(59:7)
TG(43:1)
TG(51:0)
TG(44:1)
TG(54:6)
TG(58:6)
TG(62:5)
TG(53:0)
TG(55:2)
TG(45:1)
TG(53:7)
TG(52:6)
TG(55:1)
TG(46:4)
TG(60:10)
TG(44:2)
TG(48:7)
TG(57:6)
TG(62:12)
TG(64:7)
TG(62:6)
TG(42:2)
TG(43:2)
TG(61:4)
TG(46:3)
TG(44:0)
TG(52:10)
TG(43:3)
TG(57:7)
0.0
0.2
0.4
0.6
0.8
MeanDecreaseGini
